# Persistence of plastic debris and its colonization by bacterial communities after two decades on the abyssal seafloor

**DOI:** 10.1038/s41598-020-66361-7

**Published:** 2020-06-11

**Authors:** S. Krause, M. Molari, E. V. Gorb, S. N. Gorb, E. Kossel, M. Haeckel

**Affiliations:** 1 GEOMAR Helmholtz Centre for Ocean Research, Kiel, Germany; 2 HGF-MPG Joint Research Group on Deep Sea Ecology and Technology, Max Planck Institute for Marine Microbiology, Bremen, Germany; 3 Zoological Institute, Christian-Albrechts-University, Kiel, Germany

**Keywords:** Biodiversity, Marine biology, Marine chemistry, Water microbiology

## Abstract

The fate of plastic debris entering the oceans is largely unconstrained. Currently, intensified research is devoted to the abiotic and microbial degradation of plastic floating near the ocean surface for an extended period of time. In contrast, the impacts of environmental conditions in the deep sea on polymer properties and rigidity are virtually unknown. Here, we present unique results of plastic items identified to have been introduced into deep-sea sediments at a water depth of 4150 m in the eastern equatorial Pacific Ocean more than two decades ago. The results, including optical, spectroscopic, physical and microbial analyses, clearly demonstrate that the bulk polymer materials show no apparent sign of physical or chemical degradation. Solely the polymer surface layers showed reduced hydrophobicity, presumably caused by microbial colonization. The bacterial community present on the plastic items differed significantly (p < 0.1%) from those of the adjacent natural environment by a dominant presence of groups requiring steep redox gradients (*Mesorhizobium*, *Sulfurimonas*) and a remarkable decrease in diversity. The establishment of chemical gradients across the polymer surfaces presumably caused these conditions. Our findings suggest that plastic is stable over extended times under deep-sea conditions and that prolonged deposition of polymer items at the seafloor may induce local oxygen depletion at the sediment-water interface.

## Introduction

Since the start of mass production of polymers in the 1930s plastic material is seemingly indispensable for the global economy, with an annual world-wide production of 359 Mt in 2018^1^. In Europe the largest fraction (39.9%) of plastic is used for the short-term packaging of products and food^1^. Currently, 5–13 Mt of plastic debris are believed to enter the oceans every year^2,3^, but only a few hundreds of thousand tons have been reported for the surface oceans^4,5^. While this discrepancy has been explained by various processes, such as constant export of plastic debris from the surface to the seafloor, fragmentation into small microplastic particles evading current monitoring surveys, or accumulation at shorelines^4,6^ it primarily emphasizes our knowledge gaps about the horizontal and vertical transport processes of plastic debris, particularly its associated time scales.

The pollution of the marine realm by plastic debris is of global relevance, resulting from the low chemical reactivity and consequential long-lasting rigidity of the synthetic polymers against degradation. This feature allows for long-distance travel and prolonged residence time in the marine environment. Currently, more than 60% of all debris present in the oceans is plastic with increasing trend so far^7^. This tendency is also proposed by a recent numerical modeling study indicating that the plastic pollution within the Great Pacific Garbage Patch (GPGP) is increasing exponentially^5^.

In addition to acrylic components, which are negatively buoyant in seawater, numerous plastic components exhibit positive buoyancy and hydrophobicity when entering surface waters^8,9^. In fact, about 60% of all produced plastic has a lower density than seawater^8^, since it is composed of the polymers polyethylene and polypropylene. However, after a period of weeks to months buoyant plastic can start to sink due to biofouling and particle attachment^10^. In addition, it can be speculated that plastic waste entering the ocean can be associated with other sorts of waste, which may cause immediate sinking. Wind-driven down-welling may further accelerate sinking of plastic items. As a consequence, a large fraction of the introduced plastic items will ultimately accumulate at the seafloor^9,11^.

With regard to the fate of plastic in the marine realm polymer degradation and potential mineralization pathways are of utmost importance. The degradation, here defined as decrease in molecular weight of a polymer, can principally be achieved via abiotic and biotic processes. Mineralization refers to the complete degradation of polymers to organic acids, CO_2_, CH_4_ and H_2_O^12^. In the marine environment abiotic processes facilitate initial plastic degradation. Among these, exposure to UV-radiation at the sea surface is the most important factor^7,13^ inducing photo-oxidation and subsequent cleavage of polymer chains^14^. However, due to their chemical composition, the widely used polymers polypropylene (PP) and polyethylene (PE) are considered to be rather resistant to photo-oxidative degradation^15^. In addition, various plastic products include additives acting as effective UV-stabilizers^16^. Since photo-oxidation is an autocatalytic process, it can continue without the exposure to UV-light as thermo-oxidative degradation in the presence of oxygen at Earth surface temperatures^8^. Because 90% of the UV-radiation is absorbed in the upper 50 m of the water column^17^, a rapid withdrawal of plastic debris from the ocean surface obviously limits the impact of photo-degradation, thus leading to prolonged polymer stability. Another physico-chemical degradation mechanism relevant in the marine realm is passive hydrolysis. However, the degradation rate of this process is extremely slow at Earth surface temperatures and strongly dependent on the type of polymer^7,18^.

Since plastic is largely composed of carbon compounds its role as a potential electron donor for anabolic processes of marine microbial communities is currently in the focus of intensified research. Recent studies showed that organic carbon compounds were leached from various types of plastic immersed in water, which in turn stimulated microbial growth under laboratory conditions^19,20^. However, the susceptibility of the polymers to biodegradation varies among the different types, depending on molecular weight, the presence of functional groups and additives composition. In addition, the exposure conditions, e.g. light intensity, temperature and pH, influence the degradation rate to a large extent^21^. Under marine temperature conditions, microbial degradation rates are expected to be several orders of magnitude slower compared to photo-oxidation^8^. Although photo-oxidative degradation can reduce the average molecular weight of plastic oligomers to 10^3^–10^4^ g mol^−1^ it appears to be less effective than microbial degradation, which has been observed to occur at about 500 g mol^−1^ ^8^. In contrast, a recent study indicates that the betaproteobacterium *I. sakaiensis* 201-F6, isolated from a plastic recycling site, is able to utilize polyethylene terephthalate (PET) as their sole carbon source when grown at 30 °C under non-marine laboratory conditions^22^. Microbially mediated plastic degradation has also been reported for *Actinomycetes* and fungi^21^. To what extent these findings are relevant for natural marine settings is to be deciphered yet.

For decades, the negative effects of plastic debris on marine animals via ingestion^23–25^ or entanglement^26,27^ have been reported. Besides these immediate harmful effects on eukaryotes, persisting plastic debris represents also large quantities of habitable substrate suitable for the colonization by prokaryotic microbial organisms.

During recent years a growing interest to decipher marine microbial communities specifically present on plastic surfaces has developed^28–30^. These intensified research efforts are required to constrain the ecological impacts on natural marine environments, such as seafloor habitats, subjected to plastic polymers and the associated microbial community. Also, the timescales of potential physicochemical and microbially mediated plastic degradation and remineralization are of prime environmental concern.

The present study reports on the morphological, physical, and chemical properties as well as the surface-associated microbial community of plastic debris retrieved from the seafloor in the tropical southeastern Pacific Ocean. The obtained polymer samples represent a unique data set, as the date of introduction to the marine environment could be reliably narrowed down to 1989–1996. As the duration of exposure to the marine environment is usually unknown for the vast majority of retrieved plastic items, the present study results represent, to our knowledge, the first data set reliably integrating the fate and ecological function of plastic over a time interval of more than two decades under natural marine deep-sea environmental conditions.

## Results

### Cell abundance

The initial stereomicroscopic inspection of the plastic samples showed no settlement of larvae or colonization by sessile eukaryotic organisms. Epifluorescence microscopy revealed homogeneous coverage of all plastic samples by adherent microbial biofilms. The mean cell density of the 5 different polymer samples ranged from 0.5 × 10^6^ (SD ± 0.1 × 10^6^) cells cm^−2^ to 2.4 × 10^6^ (SD ± 0.7 × 10^6^) cells cm^−2^ (Table 1). Highest average cell abundances were found on the surfaces of the plastic bag surface not in contact with the metal can (“plastic bag-no metal”) and the outside of the lid belonging to the curd box (“lid-outside”), respectively. The remaining samples showed cell densities that were a factor of two to three lower. However, as cell abundances varied well within the same order of magnitude, a preferential plastic type for microbial settlement is not apparent.

**Table 1:** Mean cell densities of the different plastic samples surfaces.

	Plastic bag-no metal	Plastic bag metal	Lid-inside	Lid-outside	Curd box
cells cm^−2^ (×10^6^)*	2.4	0.6	0.5	1.4	0.5
SD	0.7	0.1	0.1	0.7	0.1

*Mean value of five replicates. For each replicate 30–67 individual microscopic.view fiels of 6100 μm2 each were counted.

### Microbial community analysis

All diversity indices were significantly lower in plastic samples (P1–4) than in sediments and nodules samples (C1–3, R1–3, N1–3) (ANOVA: H_0_ F_(3,12)_ =53.92 and p < 0.0001, H_1_ F_(3,12)_ = 106.6 and p < 0.0001, H_2_ F_(3,12)_ = 229.8 and p < 0.0001, Chao1 F_(3,12)_ = 47.1 and p < 0.0001; Table 2).

**Table 2:** Bacterial number of 16 S rDNA gene sequences and diversity indices for samples investigated. Indices were calculated without singletons. Numbers of identified phyla, classes, orders, families, and genera are given.

Stations	ID	Sequences n.^a^	Sequences n.^b^	H_0_	H_1_	H_2_	Chao1^c^	sd	Phyla n.	Classes n.	Orders n.	Families n.	Genera n.
SO242/2_198	P1	59725	56816	194	4	2	186	7	18	38	73	90	115
SO242/2_198	P2	60940	52948	3305	349	37	3024	59	31	75	141	216	307
SO242/2_213	P3	63120	58286	1615	61	13	1484	43	31	73	139	210	291
SO242/2_213	P4	39302	27076	3079	460	54	2982	41	30	72	140	206	292
SO242/2_146	C1	225781	163892	12895	1977	419	9518	216	42	103	195	301	411
SO242/2_146	C2	155396	110338	11348	2006	437	9048	167	43	100	191	288	401
SO242/2_146	C3	116017	101752	11336	2101	463	9163	197	40	98	190	289	407
SO242/2_208	R1	77424	65705	9616	2499	663	8403	130	44	99	191	292	407
O242/2_208	R2	58575	45618	8320	2332	617	7655	125	41	91	181	273	369
SO242/2_208	R3	59503	40382	7940	2385	646	7417	114	42	96	184	279	373
SO242/2_198	N1	313418	238125	8567	846	193	6053	168	37	91	176	271	367
SO242/2_194	N2	209563	159747	9434	1267	289	7097	202	39	91	182	280	376
SO242/2_198	N3	220364	173313	8186	866	200	6049	150	38	90	173	274	368

H_0_: number of OTUs; H1: exponential Shannon; H2: inverse Simpson; Chao1: estimated richness.

^a^After the merging of forward and reverse reads.

^b^After removal of singletons and unspecific sequences (see M&M for details).

^c^Calculated with 100 sequence re-samplings per sample on the smaller dataset (N2 = 27076).

sd: standard deviation.

In the plastic samples, the estimated richness (Chao1) and abundance-based coverage estimator (H_2_) were on average 4 and 20 times lower than in the sediments, respectively, and 3 and 9 times lower than in the nodules, respectively. Also, the composition of bacterial communities (based on OTUs presence/absence) differed significantly between the plastic samples, sediments and nodules (PERMANOVA: R^2^ = 0.417, F_3,12_ = 2.15, p < 0.001). The plastic samples were more heterogenic, sharing only 19% of OTUs within the group (i.e. P2, P3 and P4), and with sample P1 that did not share OTUs with any other sample. The OTUs shared within sediment samples and within nodules samples were 39% and 30%, respectively. Only 6% of OTUs retrieved in P2, P3, and P4 were shared with sediments and nodules samples, whereas the latter two groups shared 20% of OTUs (Fig. S1).

**Figure 1:**
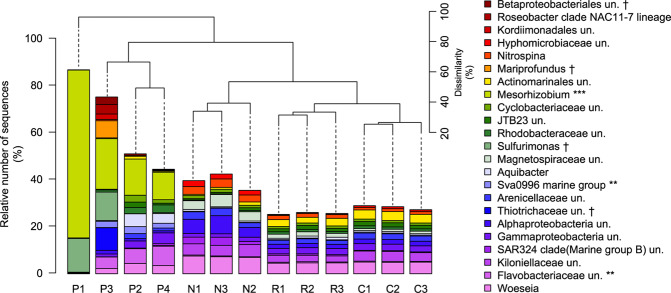
Bacterial dominant genera (cut-off ≥2%) for plastic material (P1–4), manganese nodules (N1–3), reference (R1–3) and DISCOL sediments (C1–3). Cluster on top of bar plot showed dissimilarity in community structure, as defined by Bray-Curtis dissimilarity index based on Hellinger-transformed OTU abundance data. Stars show the genera highly abundant in plastic samples (ALDEx2: glm). P1: curd box; P2: curd box lid; P3: plastic bag-metal; P4: plastic bag-no metal; un.: unclassified; **glm adjusted *p* < 0.01; ***glm adjusted *p* < 0.001; ^†^exclusively present on the plastic samples.

**Figure 2:**
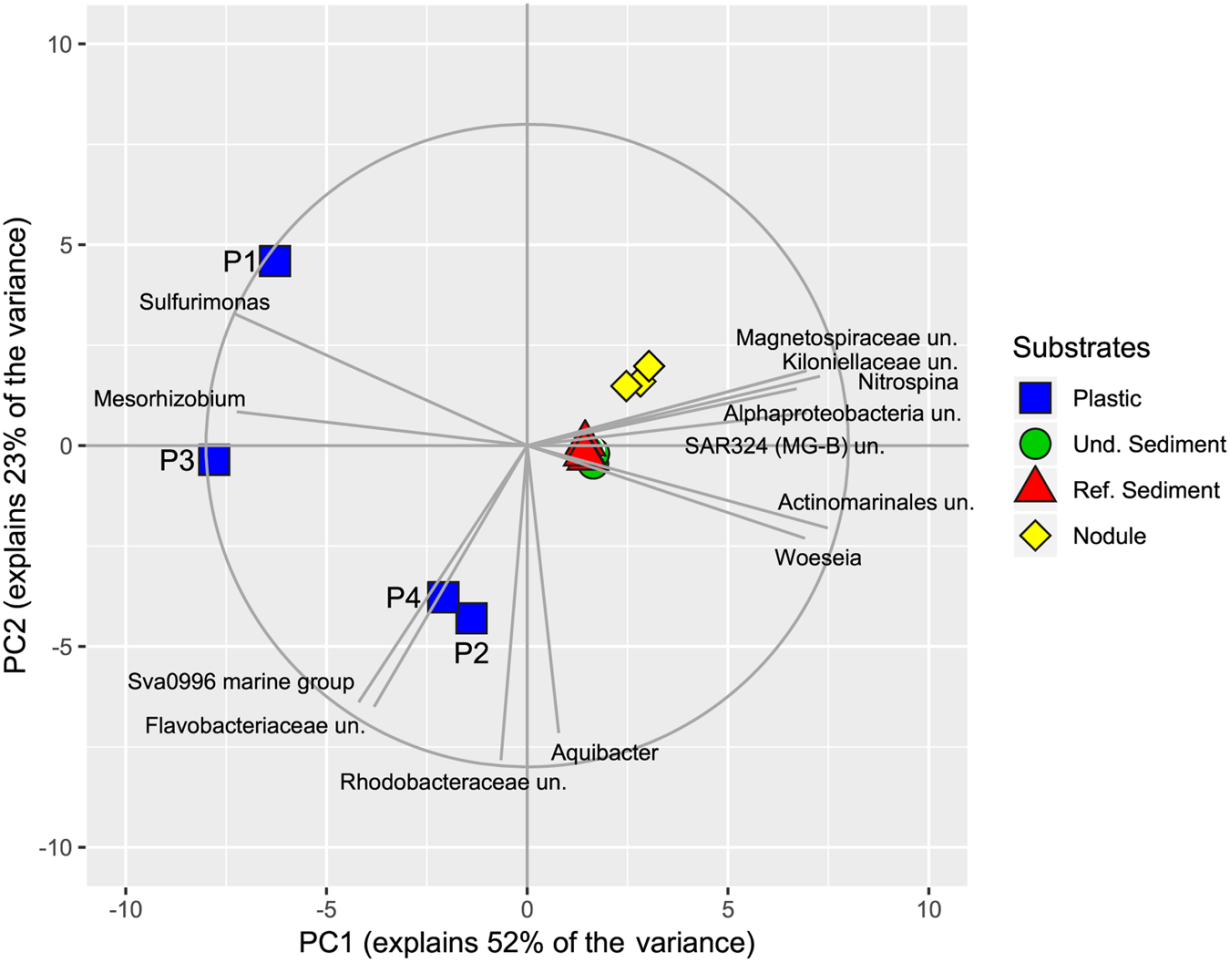
Two-dimensional principal component analysis (PCA) for bacteria community structure at genus level resolution. Hellinger-transformed genus abundance data was used for the PCA. Only genera with Pearson correlation coefficient ≤ −0.75 or ≥0.75 were plotted. The circle indicates a Pearson correlation coefficient of 1. Un.: unclassified; Und.: undisturbed; Ref.: reference.

Each substrate hosted a specific bacterial community (PERMANOVA: R^2^ = 0.569, F_3,12_ = 3.96, p < 0.001), with highest heterogeneity present in the group of plastic samples (Figs. 1 and 2).

Among the most abundant bacterial genera (contribution to total number of sequences ≥2%), *Sulfurimonas*, *Mariprofundus*, unclassified Thiotrochaceae, and Betaproteobacterales were exclusively present in plastic samples. Besides *Mesorhizobium*, the Sva0996 marine group, and unclassified Flavobacteriaceae were highly abundant in plastic samples, while absent or rare in sediment and nodule samples (ALDEx2: ANOVA adjusted p < 0.01 and KW adjusted p < 0.05). Differences in relative contribution of *Sulfurimonas* and *Mesorhizobium* were the major drivers for the differentiation of P1 and P3 community structure, whereas *Aquibacter*, unclassified Rhodobacteraceae, Sva0996 marine group, and unclassified Flavobacteriaceae were mostly responsible for differences in community structure of P4 and P2 (Figs. 1 and 2). The most abundant bacterial genera in sediments and nodules included: *Woesia*, unclassified Kiloniellaceae, unclassified Actinomarinales, unclassified SAR324 (Marine group B), unclassified Alphaproteobacteria, unclassified Magnetospiraceae, and *Nitrospina*.

### Plastic material properties

Applying Raman spectroscopy revealed that the curd box consisted of polystyrene (PS) (Fig. S2). The coating of the inner lid side appeared to be polyethylene terephthalate (PET). Unfortunately, the coating of the lid outside could not be assigned to any known plastic polymer since the spectra were dominated by contributions from pigments of the colored print on the lid. The Raman spectrum for the retrieved plastic bag was virtually identical to that of the polyethylene (PE) standard material used. Comparing the spectra to the modern reference material showed no obvious differences suggesting no obvious physico-chemical or microbial deterioration. Minor deviations in the Raman spectra between sample and reference material (Fig. S2B, wavenumber 700–800 cm^−1^) appear to result from differences in additives composition and differences in crystallinity.

**Figure 3:**
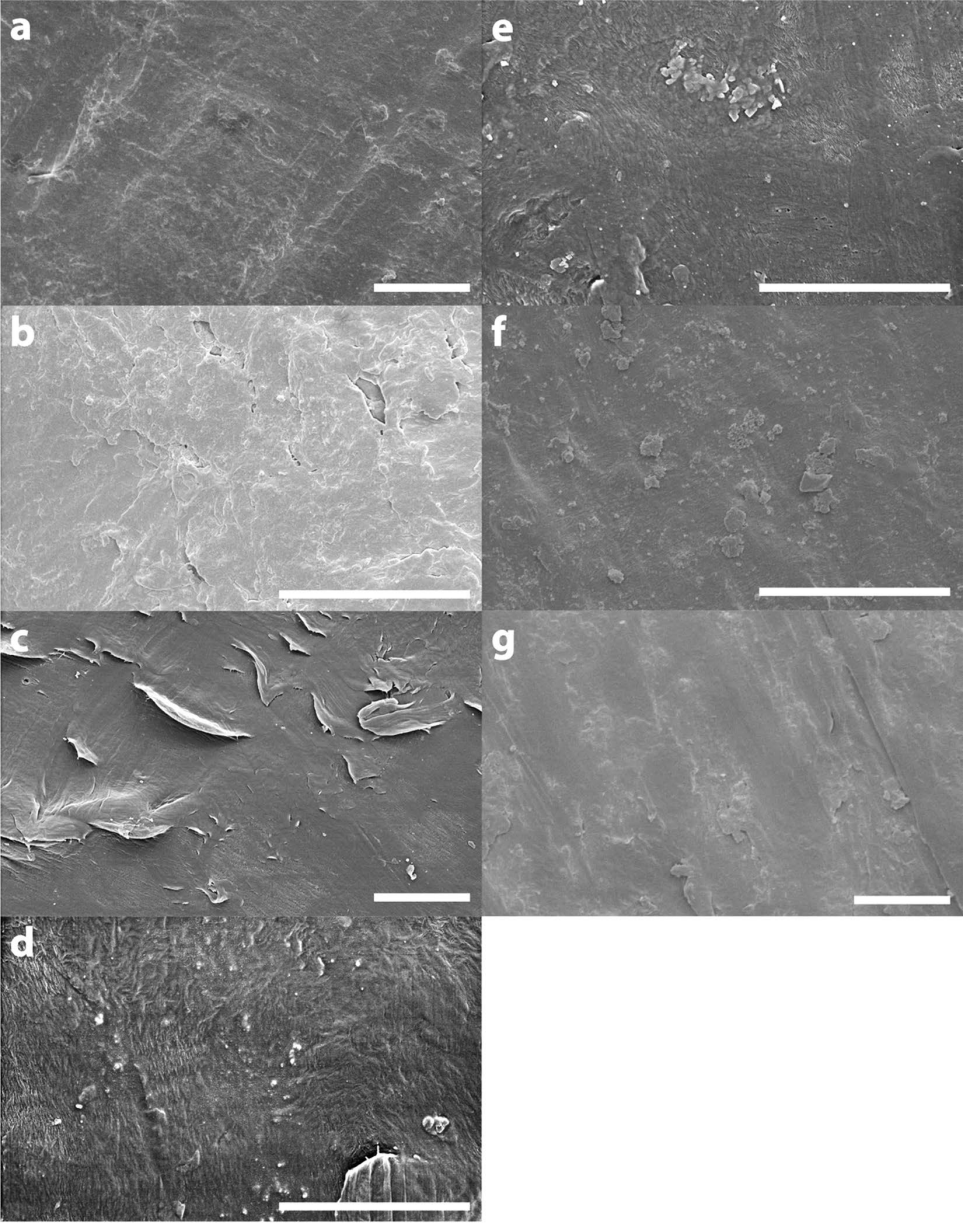
SEM images of the cleaned plastic sample surfaces and reference material. (**a**) curd box sample; (**b**) curd box reference; (**c**) lid sample; (**d**) lid-reference; (**e**) plastic bag-no metal; (**f**) plastic bag-metal; (**g**) plastic bag reference material. Scale bars indicate a length of 5 µm.

In addition to the chemical analyses also the surface structures of the plastic samples and the reference material was compared using SEM (Fig. 3).

All apparent surface defects, as irregular shallow cavities (Fig. 3a,b) or rough textures (Fig. 3c–g), were observed on the samples, as well as on the reference material. Consequently, the defects seem to be primarily a result of the production methods applied. As no principal differences in surface structure between any reference material and corresponding samples were identified, the latter appeared to represent original, unaltered topography features.

### Wettability of the surfaces

**Figure 4:**
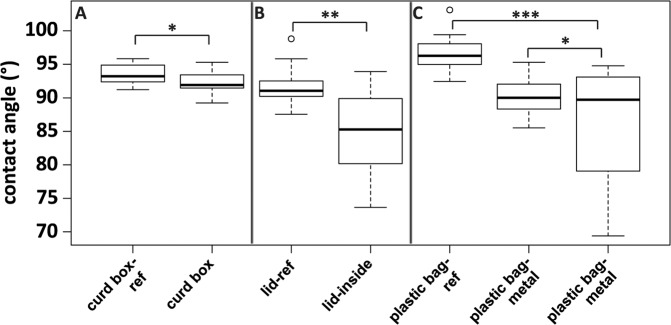
Box-Whisker-plots of contact angles of water droplets on cleaned SO242 plastic samples and reference material (n = 15, each). Brackets indicate corresponding samples and reference material for mean value comparisons (A, B t-test; C ANOVA and Tukey-test). *p ≥ 0.05, **p ≤ 0.001, ***p ≤ 0.0001.

To test a potential change of the plastic surfaces’ wettability (hydrophobicity), contact angle measurements using water droplets were carried out (Fig. 4).

The curd box and respective reference material showed similar spreads in contact angle values (Fig. 4A) with no significant difference of the mean values (t-test, *p* = 0.075). In contrast, the lid inside was characterized by a significantly lower mean value than the corresponding reference (t-test, *p* = 0.0008). For the plastic bag-samples, a highly significant difference between the reference material and the two obtained samples (plastic bag-metal and plastic bag-no metal) was observed (1-way ANOVA, *F* = 12.9, *p* = 4.5 × 10^−5^), while the means of the two plastic samples showed no significant difference (post-ANOVA Tukey-test, *p adjusted* = 0.11).

## Discussion

### Plastic deposition

The information printed in German language on the garbage items (curd box and beverage can in the plastic bag) analyzed for the present study provide evidence that the disposal is associated to the previous RV SONNE research cruises in the DEA carried out between 1989 and 1996 (see “Materials & Methods” section for details and a discussion on the uncertainty). The fact that the debris was deposited largely within or in close proximity to the DEA indicates a rather fast sinking velocity excluding extended horizontal drifting and exposure to air, UV light and waves at the sea surface. The ocean mean surface current in the study area is directed towards the southwest and typically exceeds a velocity of 10 cm s^−1^^31^, indicating a horizontal plastic drift of several kilometers per day. As the study area covers 10.8 km^2^ an extended retention period at or close to the sea surface would have resulted in the drifting of the plastic debris out of the study area. The densities of the two retrieved polymer types polyethylene and polystyrene generally range between 915 and 1040 kg m^−3^^32^, while a mean density of approximately 1027 kg m^−3^ characterizes standard surface Ocean water^33^. The individual plastic items would either stay afloat or sink at a rather low velocity, also resulting in considerable horizontal displacement prior to any potential deposition at the seafloor. Therefore, the plastic items may have entered the ocean in combination with other, denser waste objects resulting in a rather fast sinking velocity. Thus, we assume the deposition at the seafloor may have occurred within a matter of hours to days rather than weeks and that the polymers were subjected primarily to deep-sea conditions between discarding and retrieval, but have not been exposed to UV-light and wave action at the sea surface for prolonged times. Although lacking direct comparability with additional plastic samples, the two items studied contribute to the, to date, very small amount of deep-sea plastic samples with a rather reliable long-term deposition time. To our knowledge, the items analyzed in the present study represent the first data obtained for age-constrained plastic samples, thus representing an important reference point for future studies.

### Bacterial community differences

Given the fact that only two plastic items were retrieved, one can argue that the observed differences in bacterial community composition of the plastic samples and the natural occurring habitat types are simply object-specific artifacts, which are not representative for the habitat type. However, the two plastic objects differed in polymer type as well as in form factor. Nonetheless, both items showed a higher degree of similarity in bacterial community composition amongst each other than to the two natural habitat types sediment and Mn-nodule. These results indicate that the bacterial community composition on the two plastic items was not object-specific but rather a consequence of the plastic-induced diffusion barrier. As this physical feature is inherent to most polymer debris items, it can be assumed that the bacterial community associated to other plastic objects at the seafloor in the study area developed in a similar manner as the two retrieved objects.

All obtained plastic samples were characterized by a microbial biofilm covering the entire surface, regardless of the polymer type. Other studies already confirmed that initial bacterial attachment and biofilm formation happens within a matter of days^10,34^. As the samples remained at the seafloor for more than two decades it can be assumed that bacterial biofilms have been covering the surfaces virtually during the entire period of deposition. Due to the extended residence time of the plastic items at the seafloor it appears very likely that the attached microbial community is primarily the result of long-term environmental forcing factors prevailing in this deep-sea environment. Consequently, a steady state of the community structure can be assumed, which is mainly affected by the slow burial of the plastic on geological time scales due to low sediment accumulation rates of 4 mm ky^−1^ in the DEA^35^. We argue that any principal difference in microbial community structure observed between the plastic deposits and the adjacent natural benthic habitats is unbiased by a potential settlement and subsequent transfer of organisms thriving exclusively in surface waters.

The mean bacterial cell density of the samples obtained during this study ranged in the same order of magnitude (approx. 10^5^ cells mm^−2^) to those reported for pelagic plastic debris from the North Pacific Gyre^36^, further suggesting the presence of a mature microbial biofilm.

The comparison of the obtained plastic samples to the natural habitats (i.e. manganese nodules and sediments) strikingly demonstrated a principal difference in the composition of the bacterial communities present (Figs. 1, 2). The average number of OTUs (after the removal of singletons and unspecific sequences) present in the plastic samples was by a factor of 4.2 to 5.8, respectively, lower compared to the natural environments. Similar results have also been reported for plastic debris from the North Sea^37^. However, as OTU number and resulting species richness strongly depend on sample size, future investigations are required to unequivocally constrain whether plastic samples principally host a reduced bacterial community with regard to the prevailing natural habitats. The four applied diversity indices (H_0_, H_1_, H_2_, and Chao1) uniformly showed lowest values for the bacterial community present on the plastic samples, caused by the dominance of individual OTUs. The overall decrease in abundance and diversity illustrates that plastic debris has to be regarded as a rather extreme habitat, with regard to the prevailing natural environment. Whether the reduced microbial diversity is plastic-specific or can also be expanded to other artificial or natural debris remains unknown and requires further research effort. However, clear evidence exists that, in contrast to other substrates, a number of hazardous chemicals are leaching from plastic debris in water for an extended period of time^38^, as they are not chemically bound^39^. Consequently, microbial communities on plastic surfaces are subjected to a long-term flux of artificial compounds with potentially adverse metabolic impact, presumably influencing the microbial surface community structure with regard to the number of present OTUs and their distribution (evenness). To date, knowledge of the metabolic impact of plastic additive leachates on microbial communities is highly fragmentary and requires a systematic research approach.

The most abundant OTU present on the plastic samples is *Mesorhizobium*, which includes numerous members carrying out denitrification and N-fixation^40,41^. Both processes require suboxic to anoxic conditions, which are not encountered within the top 10 cm of the sediment of the study area and reference sites (Fig. S3). During the SO242-2 cruise, O_2_ concentrations of ~130 µmol l^−1^ were frequently measured at the sediment-water interface, clearly demonstrating oxic conditions. Below, O_2_-concentration typically declined exponentially to zero within the uppermost 15 cm, utilized by organic matter degradation. Subsequent redox zonations in the sediments of the DEA are characterized by nitrate and manganese(IV) oxide reduction in the top 300 cm, followed by iron(III) reduction, whereas sulfate reduction is not occurring within the upper 1000 cm^35^.

The OTUs *Sulfurimonas* and *Thiotrichaceae* were also exclusively present on the plastic samples. In addition to denitrification^42^, *Sulfurimonas* is well known to carry out sulfur oxidation^43^. The family *Thiotrichaceae* also includes a variety of aerobic and anaerobic sulfur oxidizers^44^. *Thiotrichaceae* are typically encountered in areas of methane seepage and associated anaerobic oxidation of methane (AOM)^45^. This seafloor feature is clearly absent in the sampling area. The genus *Mariprofundus* was also only identified on the plastic samples. This group carries out iron oxidation as its sole energy pathway^46^. In the marine realm, this situation is met at hydrothermal vent systems, which are not present in the study area. OTUs characteristic for the plastic items also comprised *Betaproteobacteriales*. Members of this group often grow between oxic and suboxic conditions, including species that are human pathogens^47^. Consequently, plastic debris has to be considered as a potential vector for pathogenic microbes, as has also been reported for floating microplastic^48^.

In contrast, the bacterial community of the two natural environments sediment and Mn-nodules were generally dominated by the globally occurring OTUs. These include *Woesia* and Actinomarinales, which were also present in the plastic samples. The two taxa include numerous members characterized by aerobic organic matter degradation^49,50^. The SAR324 cluster was also dominant in the environmental samples. This group is regarded as mixotrophic because of their carbon fixation and heterotrophic carbon utilization^51^. In addition, the family *Kiloniellaceae* was also characteristic for the bacterial community of the natural environments, including mainly chemoheterotrophic aerobic members with the potential for denitrification^52^.

The simultaneous presence of bacterial metabolic pathways requiring fundamentally different redox conditions vividly illustrates the existence of strong chemical gradients for the plastic habitats, contradicting the natural environmental situation. From the requirements of the prevailing bacterial communities we can deduce that the plastic items acted as highly efficient diffusion barriers, potentially causative for localized reduction of O_2_ concentrations and the simultaneous accumulation of chemically reduced compounds (e.g. sulfur and iron) by establishing a strong near-surface redox gradient. The partial accumulation of reduced metal compounds on the retrieved plastic bag further supports this hypothesis.

Another important factor for bacterial adhesion to surfaces is the degree of hydrophobicity difference. The hydrophobicity of bacteria varies considerably among individual species^53^. In general, hydrophobic surfaces are preferred by bacteria with hydrophobic properties and vice versa^54^. This discriminatory effect might contribute to explain the observed difference between the natural habitats and the deposited polymers in bacterial abundance and diversity. Biofilm formation on hydrophobic surfaces might be intensified under environmental stress (e.g. nutrient starvation) as demonstrated for *Bacillus* species^55^. In addition, biofilm formation on hydrophobic plastic might also be governed by the variable potential of physiological adaptation between different microbial organisms^56^.

It can be speculated that these two factors also influence the biofilm formation and microbial community structure in deep-sea environments. According to a recent laboratory study, the attachment of terrestrial bacterial isolates with a comparatively high degree of hydrophobicity resulted in fastest degradation rates of high impact polystyrene (HIPS) as part of electronic waste after 30 days of incubation at 30 °C^57^. This observed positive correlation of hydrophobicity and plastic degradation has yet to be established for deep-sea conditions with prevailing lower temperatures.

### Polymer properties

The optical and spectroscopic analyses of the plastic samples showed no sign of obvious abiotic mechanical^58^, photo^59^- or chemical degradation as oxygen attack^60^ or hydrolysis^61^ affecting the bulk material. Minor differences between the samples and corresponding reference materials, with regards to their Raman spectra, can be explained by differences in additive compositions and by differing percentages in crystallinity.

The only parameter that differed significantly (p < 0.05) was the water contact angle between the reference material and the obtained samples. For all polymer types, the samples showed a decrease in hydrophobicity compared to the respective reference material. This systematic difference was also observed during a previous study^34^ using a newly fabricated plastic material for incubations. We can therefore assume that the decreased hydrophobicity of the plastic surfaces was primarily caused by microbial activity and is not a long-term abiotic effect. Polymer surfaces are characterized by a number of charged functional groups governing the overall hydrophobicity^62^. These functional groups might be partially removed by microbial activity reducing the polymer surface hydrophobicity. In addition, also the leaching of additives^63,64^ might contribute to the decrease of hydrophobic molecules present at the polymers surface.

The microbial alteration of the polymer surface characteristics is termed biodeterioration^65^, a process during which the chemistry of the bulk material is not changed. The microbial attachment to the plastic is usually followed by the establishment of a biofilm, facilitating cell adhesion and resilience^66^. The extracellular substances of the biofilm can act as a surfactant facilitating the exchange between hydrophilic and hydrophobic phases^65^. Within biofilms, microbial metabolic products can accumulate inducing steep chemical gradients^67^. Among the most reactive substances produced are various inorganic and organic acids^68^, which can scavenge cations from the polymer surface to form stable complexes^69^ inducing surface erosion^70^. Microbial cation removal can occur also via oxidation processes involving siderophores present in the membranes of various chemolithotrophic bacteria^71^. Another principal mechanism for polymer surface deterioration is the activity of extracellular enzymes. Among these, microbially produced lipases, esterases, ureases and proteases are suspected to induce surface erosion^72,73^. Microbial enzymatic degradation of polymers has been reported in several studies^15,21,65^. The extracellular enzymatic activity catalyzes the cleavage of polymers into shorter chain fragments, which are small enough for transfer into the microbial cell followed by mineralization^74^. However, as no optical or spectral evidence for polymer degradation was detected during the present study, we can assume that enzymatic processes are of minor importance at environmental conditions prevailing in the deep sea. Consequently, we can conclude that the plastic samples have to some degree undergone microbial surface deterioration, while polymer chain cleavage was insignificant.

### Environmental implications

The individual position of plastic parts relative to the seafloor influenced the exchange with adjacent bottom water and potentially led to the establishments of several chemical gradients spanning from fully oxidized niches with free molecular O_2_ to strongly reduced localities. This diversification of habitat chemistry explains the observed range in potentially feasible bacterial metabolic processes among the plastic samples. In addition, the apparent reduced level of bacterial community similarity among the different plastic samples, with regards to the natural habitat types, might primarily reflect variable chemical situations of individual niches, rather than principal differences in plastic properties. In contrast, the dominant OTUs of the natural habitats, as *Woesia* and Actinomarinales are generally described as having primarily an aerobic metabolism^50^.

Although differing in element and carbon flux conditions, the natural habitat types sediment and Mn-nodule displayed a higher degree of similarity among each other, than to the bacterial community prevailing on the plastic samples. Considering the scientific history of the DISCOL area, it is noteworthy to point out that the microbial community within and outside the affected area, although showing similar dominant OTUs, could be clearly separated mathematically into two distinct groups.

Considering the clear difference of the bacterial communities between the naturally occurring habitat types and the plastic samples, our study supports the idea of a “Plastisphere” with a bacterial community distinct from the adjacent natural environment^30^. Especially the presence of OTUs typically encountered in chemically reduced environments has important environmental implications. The seafloor is a major sink for microplastic (<5 mm in diameter) debris^9^ and various studies indicate that also a considerable fraction of plastic debris >5 mm is ultimately deposited in this environment^30,75,76^. Many plastic polymers are characterized by low permeability, i.e. barrier properties^77,78^, effectively reducing the passage of dissolved gases through plastic membranes. In addition, also thin coatings enhancing especially the oxygen barrier function are vastly used^79^. Consequently, plastic debris deposited at the seafloor can create strong local oxygen gradients as consumption in the sediment might exceed trans-plastic replenishment. The fact that we observed various dominant OTUs attached to our samples preferring anoxic or suboxic conditions strongly indicates that plastic debris on a deep-sea sediment surface can effectively cause long-term oxygen-depleted conditions in this otherwise fully oxic environment (Fig. S3). We can infer that plastic debris deposition in coastal environments can cause even more severe localized anoxic conditions, because here high O_2_ consumption rates are prevailing^80^.

With regards to the samples analyzed in the present study, it is apparent that the microbial deterioration pathways clearly did not affect the polymer stability after more than two decades of deposition in the deep sea. The change in surface hydrophobicity suggests that microbial deterioration was restricted to the surface layer of the plastic items. Whether a continuing microbial colonization would result in complete remineralization is still subject of debate. However, it is plausible that, if thermodynamically granted at all, degradation reactions will proceed at very slow speed. Consequently, plastic debris in the deep sea might be present over very long, i.e. geological, time scales.

In summary, plastic items deposited on the sediment have long-term environmental impacts creating artificial habitats with strong chemical gradients. Due to the polymer surface properties and chemical gradients, specific microbial communities are likely to prosper, differing from those of the adjacent natural environment. Considering prolonged accumulation of plastic debris at the seafloor, covered areas might expand jeopardizing ecosystem functioning on all trophic levels.

## Material and Methods

### Sampling area

**Figure 5:**
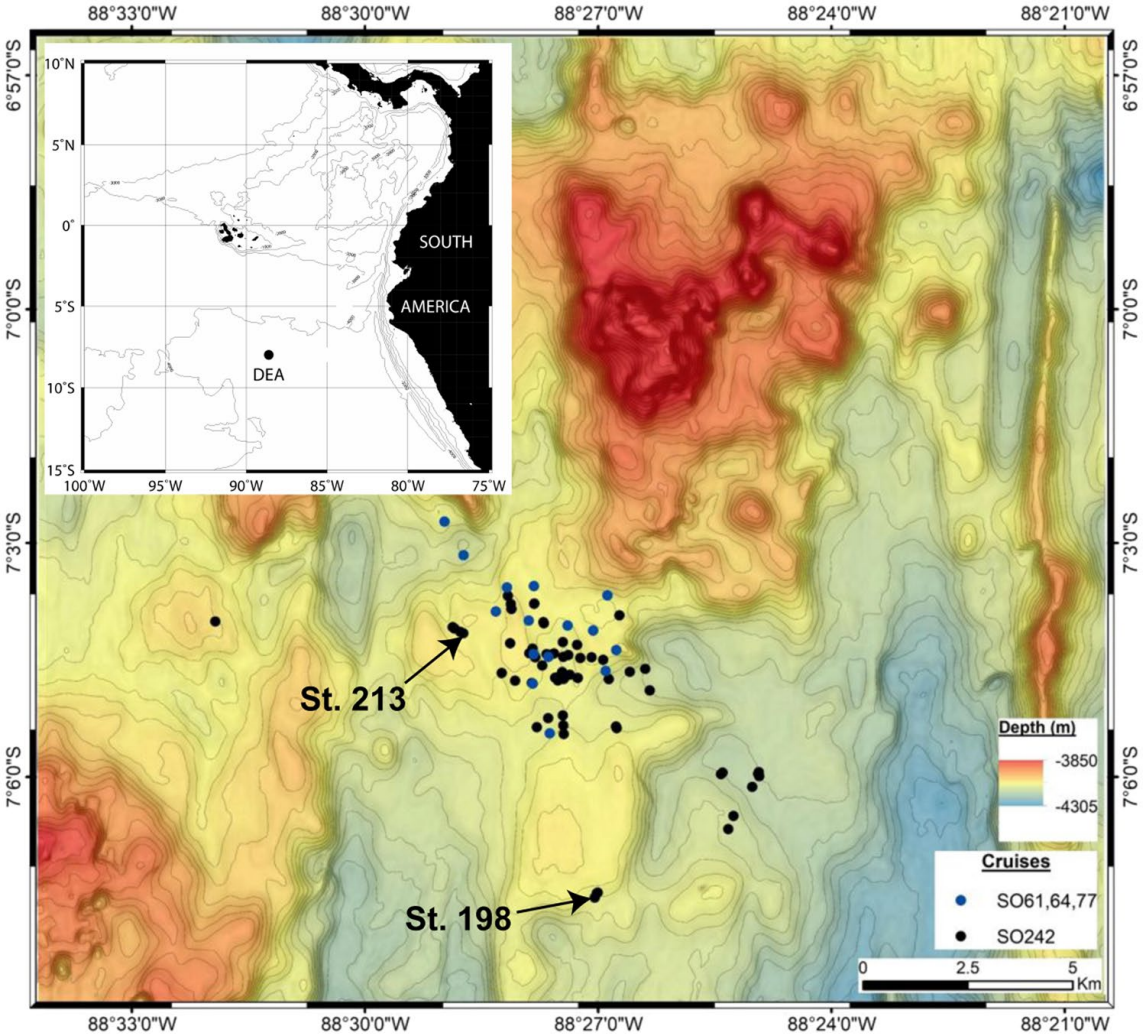
Overview (inlay) and DEA site map. The overview map was generated using the GMT 3.0 software package (http://gmt.soest.hawaii.edu/home) and the ETOPO1 bathymetry (https://www.ngdc.noaa.gov/mgg/global/). Bathymetric data of the DEA site were acquired with the EM122 multibeam on board the RV SONNE. Multibeam data were processed using the QIMERA software from QPS. The map with station positions was created in ArcGIS. The final grid cell size of the displayed bathymetric raster was set to 50 × 50 m. Contour intervals are 10 m. Blue markers show locations of waste items identified on original DISCOL OFOS images during the cruises SO61, 64, and 77 (1989–1996). Black markers indicate positions of waste items identified during SO242. Arrows denote the two positions of plastic waste items investigated in the present study.

The plastic items were collected during the second leg of the research cruise SO242 with RV SONNE (28 August–01 October 2015). The scope of this expedition was to investigate the long-term ecological effects of the benthic impact experiment DISCOL (DISturbance and reCOLonization) that was carried out in 1989 in the Peru Basin, located in the South-east Equatorial Pacific^81^ (Fig. 5).

**Figure 6:**
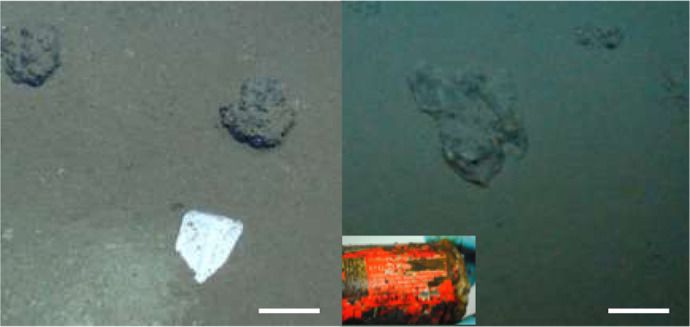
Plastic items used for the present study. Left: station 198, curd box with lid and two adjacent manganese nodules; right: station 213, plastic bag with aluminum can (inlet). Scale bars approx. 10 cm. Seafloor images taken by GEOMAR ROV KIEL 6000; Inlay image provided by Dr. M. Haeckel, GEOMAR.

The DISCOL Experimental area (DEA) covers 10.8 km^2^ of seafloor in a circular shape and is located at 7°S, 88.5°W at 4140–4160 m water depth, approximately 700 km south of the Galapagos Islands. Between 1989 and 1996, the DEA was visited four times by RV SONNE cruises: SO61 and SO64 in 1989, SO77 in 1992, and SO106 in 1996. During the SO242 cruise 32 plastic items were observed using the Ocean Floor Observation System (OFOS) and the Remotely Operated Vehicle ROV KIEL 6000. The current study focuses on the analysis of a plastic curd container, including its polymer-coated aluminum lid, and a plastic bag with an aluminum beverage can and a refreshment tissue (Alitalia airline) inside, which were picked up and retrieved during the ROV dive stations 198 and 213 (Fig. 6, Table 3), respectively.

**Table 3:** List of sampling stations during the research cruise SO242/2.

Station	SampleID	Sampling date	Latitude(N)	Longitude(N)	Depth(m)	Device	Site	Sedimentlayer (cmbsf)	Substrate
SO242/2_198	P1-curd-box	16.09.15	7.12473°	88.450°	4150	ROV	Ref.South	surface	plastic
SO242/2_198	P2-curd-box-lid	16.09.15	7.12473°	88.450°	4150	ROV	Ref.South	surface	plastic
SO242/2_213	P3-plastic-bag-metal	21.09.15	7.07943°	88.468°	4150	ROV	DEAWest	surface	plastic
SO242/2_213	P4-plastic-bag-nometal	21.09.15	7.07943°	88.468°	4150	ROV	DEAWest	surface	plastic
SO242/2_146	C1	01.09.15	7.0741°	88.464°	4130	ROV/PUC	Undist.West	0–1	sediment
SO242/2_146	C2	01.09.15	7.0741°	88.464°	4130	ROV/PUC	Undist.West	0–1	sediment
SO242/2_146	C3	01.09.15	7.0741°	88.464°	4130	ROV/PUC	Undist.West	0–1	sediment
SO242/2_194	N2	15.09.15	7.0761°	88.526°	4130	MUC	Ref.West	surface	nodule
SO242/2_198	N1	16.09.15	7.1262°	88.450°	4146	ROV	Ref.South	surface	nodule
SO242/2_198	N3	16.09.15	7.1262°	88.450°	4146	ROV	Ref.South	surface	nodule
SO242/2_208	R1	15.09.15	7.0761°	88.526°	4130	MUC	Ref.South	0–1	sediment
SO242/2_208	R2	15.09.15	7.0761°	88.526°	4130	MUC	Ref.South	0–1	sediment
SO242/2_208	R3	15.09.15	7.0761°	88.526°	4130	MUC	Ref.South	0–1	sediment

The plastic bag part in contact with the aluminum can showed a thin, black crust after retrieval. This crust turned rusty, orange when exposed to air, indicating that reduced iron was oxidized. Other parts of the bag were free of this crust. The colored coating of the curd box lid outside was partly detached or missing, potentially biasing surface properties. Therefore, this side of the lid was excluded from chemical and physical surface analyses. For microbial community investigation both sides of the lid were sampled together as bulk. From the production information on the curd box lid, 5-digit postal number (introduced in Germany in 1990) and the manufacturer “Bremerland Nordheide Molkerei eG” (bought in 1999 by “Nordmilch eG”) we could constrain the potential disposal period to the two RV SONNE cruises carried out in 1992 or 1996. The beverage “Coke” can in the plastic trash bag is a special edition produced in Germany for the “Davis Cup” in December 1988 and showed an expiry date in 1990. Therefore, the plastic trash bag with the can and a refreshment tissue inside was very likely introduced during one of the RV SONNE cruises carried out in 1989. As dates of production and introduction to the marine environment were rather close and both items were found at the seafloor inside the working area of the respective RV SONNE cruises, a prolonged aging of the polymers at aerial conditions and the sea surface is unlikely. While this information indicates strong evidence for an exposure time of 26 years for the can and 19–23 years for the plastic bag, it needs to be acknowledged that the investigated items were not deliberately placed at the seafloor and as a consequence they could potentially originate from other sources introducing some uncertainty for the time of deposition.

From now on, the following terminology will be applied on the different samples described above: “curd box”, “lid-inside”, “lid-outside”, “plastic bag-metal” and “plastic bag-no metal”, respectively. For the comparison of microbial communities natural samples inside the DEA, including sediment and manganese nodules, were obtained with a multi-corer (MUC) and ROV using push cores (PUC), respectively (Table 1). Additionally, reference sediment outside the DEA was sampled with a multiple corer (MUC).

### Sampling procedures

Sediment, manganese nodules, and plastic items were sampled with two different devices: the remotely operated vehicle ROV KIEL 6000 and a multiple corer (MUC). Acryl-glass tubes (push cores) were used for ROV-based sediment sampling. Each push core had an overall length of approximately 27 cm and an inner diameter of 0.75 cm. A one-way valve was mounted on the top opening of each core allowing for vacuum build-up during core retraction. Using the robotic arms of the ROV, up to approximately 75% of each core was pushed vertically into the sediment. After 1–2 minutes the core was extracted and carefully placed on a conical rubber stopper inside a core box mounted on the ROV. Plastic items or Mn-nodules were picked up from the seafloor using the robotic arms of the ROV. For transport the retrieved items were placed in the ROV’s drawer to avoid extended movement during the remaining dive. After ROV recovery all samples were immediately transported to the cold-room (approx. 4 °C) for further processing.

In addition, also the MUC was used for sediment and manganese nodule sampling. This device was equipped with 12 transparent acryl-glass tubes, each approximately 62 cm in length with an inner diameter of approximately 9.4 cm. Positioning beacons were attached to the cable 50 m above the MUC. Before landing on the seafloor the MUC was kept on a fixed wire length for 1–2 minutes to relieve any wire twisting. Subsequently, the MUC was lowered with a speed of 0.3 m s^−1^ until landing on the bottom. Upon contact with the seafloor the cylinders were slowly driven into the seafloor. After 1–3 minutes the MUC was heaved initiating automatic closure of the tubes’ open ends. Immediately after recovering the MUC the tubes were transported to the cold-room (approx. 4 °C) for further processing.

### Sample treatment

From the different plastic items, subsamples were cut with a sterilized pair of scissors. One fraction was immediately transferred into sterile plastic bags without further treatment and frozen at −20 °C until further use. Another fraction was rinsed with ultra-purified water, transferred into sterile plastic bags and also frozen at −20 °C. For diagnostic epifluorescence microscopy a third fraction was fixed in 4% formalin for two to four hours, followed by carefully repeated rinsing with sterilized phosphate-buffered saline (PBS). Subsequently, these plastic items were transferred into sterile 50-ml centrifuge tubes with a 1:1 ethanol/PBS solution and also stored at −20 °C.

The Sediment cores were sliced on board in the cold-room, and aliquots of sediment were stored at −20 °C for DNA extraction. The manganese nodules were gently rinsed with 0.22-μm filtered cold bottom seawater to remove adhering sediment, stored in sterile plastic bags at −20 °C and crushed in the home lab before DNA extraction.

### Microbial community analysis

#### Cell counts

Prior to any microbial cell enumeration, all plastic samples were rinsed three times with sterile PBS to remove all microbes not firmly attached to the polymer surfaces. For the identification of potential colonization by eukaryote organisms, all plastic samples were inspected using a Zeiss Stereomicroscope with a maximum magnification of factor 100. Direct microbial cell counting was carried out on 5 randomly selected subsamples (approx. 10 × 10 mm^2^), which were cut out with sterile scalpels from each of the plastic sample types. All subsamples were coated with a 0.1% agarose solution and dried at 37 °C for 15 minutes. Subsequently, each sample was stained with 100 µl of 4′,6 -diamidino-2-phenylindole (DAPI) (1 µg ml^−1^) for 10 minutes at room temperature in the dark. After staining, samples were washed in ultra-purified water for 10 minutes in the dark, followed by short dipping in ethanol (97%). After complete ethanol evaporation, samples were mounted on microscopy glass slides applying 20 µl of the anti-fading agent CITIFLUOR AF-1 before addition of the cover slip. Cell counting was carried out with a Zeiss AxioImager.M2 Epi-fluorescence microscope equipped with a Zeiss AxioCam MRm Rev 3 using a DAPI filter set. Images were obtained with the Zeiss software package ZEN 2.3. For each sample, a minimum of 10^3^ DAPI-stained cells in 30 to 67 independent microscopic fields of 6100 µm^2^ each were counted at a magnification of 1,000.

#### DNA extraction

For comparison, microbial DNA was extracted not only from the plastic samples, but also from adjacent surface sediment and manganese nodules, as well as from sediment of a reference station. For DNA extraction, original plastic samples were cut in small pieces and manganese nodules were crumbled, avoiding any potential risk of contamination. Total DNA was extracted from 0.5 g plastic material, crushed nodule and sediment (homogenized 0–1 cm layer) with the FastDNA SPIN Kit for Soil (Q-BIOgene, Heidelberg, Germany), applying the manufactory instruction with the following modifications: i) after adding the Sodium Phosphate Buffer and the MT Buffer the sample was incubated for 15 min. at room temperature; ii) after FastPrep homogenization the sample was incubated for 10 min. at 65 °C; iii) after adding the binding matrix, the sample was inverted for 4 min. and left for settling for another 4 min. before 550 µl of the supernatant was discarded; iv) the final DNA on the binding matrix was solved two times in 30 µl TE buffer and incubated at 55 °C for 5 min. before centrifugation. A microplate spectrometer (Infinite 200 PRO NanoQuant, TECAN Ltd, Switzerland) was used for DNA quantification.

#### Amplicon sequencing

Amplicon sequencing was done at the CeBiTec laboratory (Centrum für Biotechnologie, Universität Bielefeld) on an Illumina MiSeq machine (Illumina, San Diego, CA). For the 16 S amplicon library preparation we applied a two-step PCR protocol following the procedure recommended by illumina (16 S Metagenomic Sequencing Library Preparation, Part # 15044223, Rev. B). The hypervariable V3–V4 region of the bacterial 16 S rDNA was sequenced using the bacterial primers S-D-Bact-0341-b-S-17 (5′-CCTACGGGNGGCWGCAG-3′) forward and S-D-Bact-0785-a-A-21 (5′-GACTACHVGGGTATCTAATCC-3′) reverse^82^. The amplicon library was sequenced with the MiSeq v3 chemistry, in a 2 × 300 bases paired device and with a number of reads per sample >50,000. Raw paired-end reads were primer-trimmed using cutadapt^83^. For quality trimming, a sliding window of four bases and a minimum average quality of 15 was applied in trimmomatic v0.32^84^, and the reads were merged using PEAR v0.9.5^85^. Clustering into operational taxonomic units (OTUs) was done with the swarm algorithm using default parameters (v2.0^86^). One representative sequence per OTU was taxonomically classified with SINA (SILVA Incremental Aligner; v1.2.11; Silva reference database release 132) at a minimum alignment similarity of 0.9, and a last common ancestor consensus of 0.7^87^. OTUs that were classified as chloroplasts, mitochondria, Archaea, and those not classified at the domain level were excluded from further analyses. OTUs that occurred with only a single sequence in the whole dataset (i.e. singletons) were also removed (Table 2). We define the most abundant genera, as the genera with a sequences contribution of total number of sequences ≥2% (retaining only the OTUs with a relative number of sequences ≥0.1%). For microbial analyses the plastic samples were defined as P1 – curd box, P2 – curd box lid, P3 – plastic bag-metal, and P4 – plastic bag-no metal.

### Data and Statistical analysis

The first three Hill Numbers, or the effective number of species, were used to describe alpha-diversity (Table 2): species richness (H_0_), the exponential of Shannon entropy (H_1_), and the inverse Simpson index (H_2_^88^. Calculation of the estimated richness (Chao1) was based on repeated (n = 100) random subsampling of the amplicon data sets (27076 sequences), to account for differences in sequencing depth between samples. Significant differences in alpha-diversity indices between substrates (i.e. plastics, manganese nodules and sediments) were determined by analysis of variance (ANOVA), or by non-parametric Kruskal-Wallis test (KW) when ANOVA’s assumptions were not satisfied.

Beta-diversity in samples from different substrates and from the substrate in samples from different sites was quantified by calculating Bray-Curtis dissimilarity based on Hellingen transformed^89^ OTU abundances (i.e. community structure) and Jaccard dissimilarity based on a presence/absence OTU table (i.e. community composition). The latter was calculated with 100 sequence re-samplings per sample on the smallest dataset (27076 sequences), to account for differences in sequencing depth between samples. Bray-Curtis and Jaccard dissimilarities were used for hierarchical cluster analysis applying average linkage method. Differences in community structure and composition (i.e. groups returned by cluster analysis) were tested with the permutational multivariate analysis of variance (PERMANOVA^90^). Jaccard dissimilarity coefficient was used to calculate the number of shared OTUs within samples and between substrates.

The genera responsible for differences observed in bacterial communities between substrates were identified applying differential abundance analysis (ANOVA like) and Principal Component Analysis (PCA). Differentially abundant genera were detected using the R package ALDEx2^91^ at a significance threshold of 0.01 and 0.05 for Benjamini–Hochberg (BH) adjusted parametric and non-parametric (KW) p-values, respectively. Prior to PCA, 16 S rDNA based OTU abundances were Hellinger-transformed^89^, and after analysis only genera with Pearson correlation coefficient ≤ −0.75 or ≥0.75 were plotted.

All statistical analyses were conducted in R using the core distribution (version 3.3.0^92^ and the following packages: Vegan^93^, ALDEx2^91^, ggplots2^94^.

### Data Accession numbers

Clipped-trimmed reads are available on ENA Accession Number: PRJEB30517, PRJEB32680, and PRJEB33205. The data were archived using the brokerage service of GFBio.

### Material analysis with Raman spectroscopy

As the plastic items were produced more than two decades ago, it was not possible to find the exact same material for reference measurements. However, a modern curd box and an unused transparent polyethylene bag, both purchased in 2015 were used as analogues for reference measurements. Prior to any measurements, 1 × 1 cm^2^ areas were cut out from the sample and reference material. All pieces were cleaned carefully with KIMTECH wipes to remove any biofilm or lint from the surface. Subsequently, all pieces were rinsed 5 times with ultra-purified water and 3 times with ethanol (96%). Pieces were than left to dry in sterile Petri dishes at 20 °C. For analyses the plastic bag pieces were flattened without stretching and fixed to microscopy glass slides.

To identify potential chemical alteration, sample and reference material surfaces were analyzed with confocal Raman spectroscopy. For most polymers, Raman spectroscopy is not a very susceptible method for detecting chemical changes due to weathering^95^. Changes in the spectra mainly manifest themselves in the form of altered relative peak heights, which can also be caused by a normal variation of the polymer crystallinity. Strong weathering, however, leads to a significant deterioration in spectral quality and signal-to-noise ratio^96^ and would be observable in the data. The sensitivity of the Raman spectroscopy depends, among other factors, on the size of the focal spot, which stretches over several µm. Hence, Raman spectroscopy cannot detect changes in the polymer matrix, which are limited to sub-µm layers.

The analyses were carried out with a LabRAM HR800 (Horiba Jobin Yvon GmbH, Bensheim, Germany) spectrometer, equipped with a 473 nm laser with a power output of 50 mW and a 1800 grooves/mm diffraction grating. The detector entrance slit was opened to 100 µm width. Sample material was excited with a 50% attenuation filter over 5 integration cycles of 3 s duration each. The incident beams were focused onto the samples through a 100 Å- magnification objective. The confocal hole closed to 200 µm to reduce potential signals from fluorescence effects. A Si-waver was used before and after the measurements to calibrate the instrument. Multiple point measurements were carried out on each sample to check for spatial material heterogeneity.

The obtained spectra were identified using the Horiba edition of the KnowItAll Raman database (Bio-Rad, Philadelphia, PA, USA) and compared to newly fabricated reference material.

### Scanning electron microscopy surface imaging

For each sample type, two pieces of approximately 5 × 5 mm^2^ were cut out using sterile scissors or scalpels. The pieces were rinsed gently with ultra-purified water and cleaned carefully using non-fuzzy tissues. Subsequently, all samples were rinsed again with ultra-purified water and dried over night at 20 °C.

For scanning electron microscopy (SEM) all samples were carefully cleaned with ethanol (96%). After complete evaporation, samples were mounted on aluminum stubs, using double-sticky carbon plates and sputter-coated with a 10 nm thick gold/palladium layer. Surface imaging was carried out using a Hitachi S-4800 scanning electron microscope. Images were obtained at 3 kV at a working distance of 13.9 mm using the lower secondary electron emission (SE) detector.

### Contact angle measurements

Surface wettability was determined by contact angle measurements of ultra-purified water (surface tension = 72.1 mN m^−1^, dispersion component = 19.9 mN m^−1^, polar component = 52.2 mN m^−1^) on all plastic sample types using the high-speed optical contact angle measuring device OCAH 200 (DataPhysics Instruments GmbH, Filderstadt, Germany). Individual 1-µl water drops were deposited on the sample surfaces and imaged horizontally (sessile drop method). Subsequently, mathematical fitting functions (circle or ellipse fitting) were used for the extraction of the drop profile and measuring of the apparent contact angles of the drops. For each material, 15 drops were measured. The average contact angle values of the different polymers were analyzed for statistically significant differences by unpaired t-tests and analysis of variance using the R software package.

## Supplementary information

Supplementary information.

## Data Availability

The genetic datasets generated during the current study are available on ENA Accession Numbers PRJEB30517, PRJEB32680, and PRJEB33205. The remaining data generated during the current study are available from the corresponding author on reasonable request.
