# Supplementary material for: Persistence of plastic debris and its colonization by bacterial communities after two decades on the abyssal seafloor
Source: Sci Rep. 2020 Jun 11;10:9484. doi: 10.1038/s41598-020-66361-7 (PMC7289819; doi:10.1038/s41598-020-66361-7)
Supplement: Supplementary file 1 — Supplementary information. [file 41598_2020_66361_MOESM1_ESM.docx]

Supplementary Information

Persistence of plastic debris and its colonization by bacterial communities after two decades on the abyssal seafloor

S. Krause^1^, M. Molari^2^, E.V. Gorb^3^, S.N. Gorb^3^, E. Kossel^1^, M. Haeckel^1^

^1^GEOMAR Helmholtz Centre for Ocean Research Kiel, Germany

^2^ HGF-MPG Joint Research Group on Deep Sea Ecology and Technology, Max Planck Institute for Marine Microbiology, Bremen, Germany

^3^ Zoological Institute, Christian-Albrechts-University Kiel, Germany

Correspondence and requests should be addressed to S. Krause (skrause@geomar.de)

Figure S1. Hierarchical dendrogram showing Jaccard dissimilarity of bacterial community composition between substrates types (P1-P4 plastic; C1-C3 DEA sediment; R1-R3 reference sediment outside DEA; N1-N3 manganese nodules). The Jaccard dissimilarity index is based on presence/absence OTU table and calculated with 100 sequence re-samplings per sample on the smallest dataset (N2=27076 sequences). The percentage of shared OTUs is reported close to highlighted nodes.

Figure S2. Raman spectra of SO242 plastic samples and reference material. A: curd box material with polystyrole reference; B: coating of lid-inside with poly(ethylene terephthalate) reference; C: plastic bag and polyethylene reference.

Figure S3. Oxygen profiles in sediments from inside the DEA area (DEA 1, 2) and references outside (Ref 1, 2).
